# Dlx-2 and glutaminase upregulate epithelial-mesenchymal transition and glycolytic switch

**DOI:** 10.18632/oncotarget.6879

**Published:** 2016-01-11

**Authors:** Su Yeon Lee, Hyun Min Jeon, Min Kyung Ju, Eui Kyong Jeong, Cho Hee Kim, Hye Gyeong Park, Song Iy Han, Ho Sung Kang

**Affiliations:** ^1^ Department of Molecular Biology, College of Natural Sciences, Pusan 609-735, Korea; ^2^ Nanobiotechnology Center, Pusan National University, Pusan 609-735, Korea; ^3^ The Division of Natural Medical Sciences, College of Health Science, Chosun University, Gwangju 501-759, Korea

**Keywords:** Dlx-2, GLS1, Snail, epithelial-mesenchymal transition, glycolytic switch

## Abstract

Most cancer cells depend on enhanced glucose and glutamine (Gln) metabolism for growth and survival. Oncogenic metabolism provides biosynthetic precursors for nucleotides, lipids, and amino acids; however, its specific roles in tumor progression are largely unknown. We previously showed that distal-less homeobox-2 (Dlx-2), a homeodomain transcription factor involved in embryonic and tumor development, induces glycolytic switch and epithelial-mesenchymal transition (EMT) by inducing Snail expression. Here we show that Dlx-2 also induces the expression of the crucial Gln metabolism enzyme glutaminase (GLS1), which converts Gln to glutamate. TGF-β and Wnt induced GLS1 expression in a Dlx-2-dependent manner. GLS1 shRNA (shGLS1) suppressed *in vivo* tumor metastasis and growth. Inhibition of Gln metabolism by shGLS1, Gln deprivation, and Gln metabolism inhibitors (DON, 968 and BPTES) prevented Dlx-2-, TGF-β-, Wnt-, and Snail-induced EMT and glycolytic switch. Finally, shDlx-2 and Gln metabolism inhibition decreased Snail mRNA levels through p53-dependent upregulation of Snail-targeting microRNAs. These results demonstrate that the Dlx-2/GLS1/Gln metabolism axis is an important regulator of TGF-β/Wnt-induced, Snail-dependent EMT, metastasis, and glycolytic switch.

## INTRODUCTION

Metabolic changes in cancer cells support higher rates of cell proliferation and growth compared to normal cells [1–8]. Most cancer cells rely on glycolysis more than mitochondrial oxidative phosphorylation for ATP production, even in the presence of oxygen; as a consequence, they exhibit increased glucose (Glc) uptake and lactate (Lac) production [1–3]. This phenomenon, termed the “Warburg effect” or glycolytic switch, is thought to increase the availability of biosynthetic precursors for nucleotides, lipids, and amino acids required for cancer cell proliferation. Glutamine (Gln) metabolism is also enhanced in many cancers [1, 2, 4–8]. Gln metabolism refills the pool of TCA cycle intermediates (anaplerosis), which are removed from mitochondria to participate in biosynthetic reactions for lipids, nucleic acids, and proteins (cataplerosis) [9–11]. Thus, Gln anaplerosis is crucial in maintaining cancer cell growth and development. Glutaminase (GLS), which converts Gln to glutamate, is the first enzyme in Gln anaplerosis [10, 11]. There are two types of GLS in mammalian cells: the kidney type GLS1 and the liver type GLS2. GLS1 is important in cancer development and progression [12–17], and its levels are increased in many tumors, including breast and prostate cancer and hepatocellular carcinoma (HCC) tissues, compared to normal tissues [17, 18]. GLS1 shRNA (shGLS1), and the GLS-specific inhibitors bis-2-(5-phenylacetamido-1,3,4-thiadiazol-2-yl)ethyl sulfide (BPTES) and 968, reduce the growth of several cancer types in xenograft models [10, 14, 17].

Epithelial-mesenchymal transition (EMT) is essential for the initiation of metastasis, which is the most common cause of death in cancer patients [19–23]. EMT involves profound phenotypic changes, including the loss of epithelial cell polarity following reductions in levels of epithelial proteins, such as E-cadherin, and increases in levels of mesenchymal proteins, such as vimentin, that upregulate mesenchymal migration and invasion [19, 22]. EMT contributes to chemoresistance and cancer stem cell-like phenotypes. Snail, the major transcription factor in EMT, triggers metastasis in response to several oncogenic signaling pathways, including transforming growth factor (TGF)-β and Wnt, in breast carcinoma, prostate cancer, and colorectal cancer [24–28]. Recently, Snail has been shown to induce glycolytic switch, suppress mitochondrial respiration and cytochrome c oxidase activity [29], and suppress fructose-1,6-biphosphatase expression [30]. Glc metabolism may therefore regulate the induction of EMT.

Recently, we showed that distal-less homeobox-2 (Dlx-2), a homeodomain transcription factor involved in embryonic [31, 32] and tumor development [33–36], induces EMT and a glycolytic switch by increasing Snail expression [37]. Because Dlx-2 induces glycolytic switch via Snail induction, we postulated that Dlx-2 may activate other oncogenic metabolic pathways. We examined the effects of Dlx-2 on the expression of GLS1 and Gln metabolism. We also studied the impact of GLS1 knockdown on *in vivo* tumor metastasis and growth. To determine whether GLS1 and Gln metabolism influence TGF-β/Wnt/Dlx-2/Snail-induced EMT and glycolytic switch, we examined p53-dependent regulation of Snail-targeting microRNAs (miRNAs) and Snail mRNA stability. Finally, we measured levels of Dlx-2, GLS1, Snail and Snail-targeting miRNAs in human cancer tissues. These experiments clarified the role of the Dlx-2/GLS1 axis in TGF-β/Wnt-induced, Snail-dependent EMT, metastasis, and glycolytic switch.

## RESULTS

### GLS1 is induced by Dlx-2

Because Dlx-2 induces glycolytic switch by inducing Snail expression [37], we postulated that Dlx-2 may activate other oncogenic metabolic pathways.

MCF-7 cells are non-invasive luminal A subtype breast cancer cells [38]. Dlx-2 and Snail induce EMT and glycolytic switch in MCF-7 cells [29, 30, 37]. Dlx-2 mRNA levels were lower in MCF-7 cells than in HCT116, HepG2, and HeLa cells; MCF-7, MDA-MB231, and A549 cells had similar Dlx-2 mRNA levels. Under normal conditions, Dlx-2 mRNA levels in the different cell lines were as follows relative to levels in HCT116 cells, which were used as a reference cell line: 0.446-fold in MCF-7, 0.351-fold in MDA-MB231, 0.322-fold in A549, 1.389-fold in HepG2, and 1.144-fold in HeLa.

**Figure 1:**
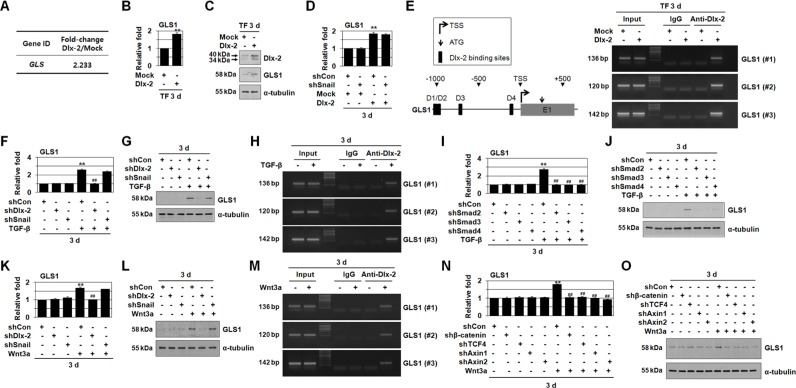
TGF-β and Wnt3a induces GLS1 expression by Dlx-2 activation **A-C.** MCF-7 cells were transfected with Dlx-2. Changes in cellular gene transcription were detected by microarray analysis (A). Fold increases in expression as compared with Mock are shown. The cells were also analyzed by real-time qrtPCR (B) and immunoblotting (C) using the indicated primers and antibodies. **p < 0.01 versus Mock. **D.** MCF-7 cells co-transfected with Dlx-2 and shSnail were analyzed by real-time qrtPCR for GLS1 expression. **p < 0.01 versus Mock. **E.** A schematic diagram of the human *GLS1* promoter regions is shown in left panel, and the 4 predicted Dlx-2 binding sites are indicated by black boxes and numbered D1, D2, D3 and D4. The ChIP-enriched DNA was amplified using primers #1, #2 or #3, which encompass the D1/D2, D3, or D4 binding sites in the GLS1 promoter, respectively. MCF-7 cells were transfected with Dlx-2 and analyzed using ChIP assays (right panel). **F, G.** MCF-7 cells transfected with shDlx-2 or shSnail and then treated with TGF-β were analyzed by real-time qrtPCR (F) and immunoblotting (G) for GLS1 expression. **p < 0.01 versus untreated, ^##^*p* < 0.01 versus shCon. **H.** MCF-7 cells were treated with TGF-β and analyzed using ChIP assays. **I, J.** MCF-7 cells were transfected with shRNA for Smad 2/3/4 and then treated with TGF-β. The cells were then analyzed by real-time qrtPCR (I) and immunoblotting (J) for GLS1 expression. **p < 0.01 versus untreated, ^##^*p* < 0.01 versus shCon. **K, L.** MCF-7 cells transfected with shDlx-2 or shSnail and then treated with Wnt3a CM were analyzed by real-time qrtPCR (K) and immunoblotting (L) for GLS1 expression. **p < 0.01 versus untreated, ^##^*p* < 0.01 versus shCon. **M.** MCF-7 cells were treated with Wnt3a CM and analyzed using ChIP assays. **N, O.** MCF-7 cells were transfected with shRNA for β-catenin, TCF4, and Axin1/2 and then treated with Wnt3a CM. The cells were then analyzed by real-time qrtPCR (N) and immunoblotting (O) for GLS1 expression. **p < 0.01 versus untreated, ^##^*p* < 0.01 versus shCon. All error bars represent SE. For all immunoblotting images, cropped blots are shown.

We examined potential metabolism-linked target genes of Dlx-2 using cDNA microarray technology (Agilent Human Genome 8x60K array, Agilent technologies, CA) and MCF-7 cells. Among ~ 42,400 genes examined on this chip, Dlx-2 upregulated several metabolic enzymes, including GLS1, PFKFB2, H6PD, and ACACB. These enzymes are involved in Gln metabolism, glycolysis, pentose phosphate pathway (PPP), and fatty acid/cholesterol synthesis, respectively, suggesting that Dlx-2 may activate several oncogenic metabolic pathways (the microarray dataset is available in GSE61009). Dlx-2 led to a 2-fold upregulation of GLS1 (Figure 1A), without affecting the expression of other Gln metabolism enzymes, including GLS2, GLUD1, GOT1/2, and ME1 (data not shown).

Most cancer cells depend on enhanced Gln metabolism for growth and survival in addition to Glc metabolism [2, 4–8]. GLS1, which converts Gln to glutamate [10, 11], is the first enzyme involved in Gln anaplerosis. Because of its importance for Gln anaplerosis, we focused on Dlx-2-induced changes in GLS1 mRNA levels, even though they only increased 2-fold in the microarray data.

Real-time quantitative reverse transcription PCR (real-time qrtPCR, Figure 1B) and immunoblotting (Figure 1C) confirmed the microarray data; Dlx-2 overexpression increased GLS1 mRNA and protein levels. Because Snail acts downstream of Dlx-2, we examined the effects of shSnail on Dlx-2-induced GLS1 expression. shSnail did not inhibit Dlx-2-induced GLS1 expression (Figure 1D), suggesting that Dlx-2 induces GLS1 expression independently of Snail. In addition, Snail overexpression had no effect on GLS1 expression (data not shown).

We further examined the effects of Dlx-2 levels on GLS1 expression using a ChIP assay. Dlx-2 homeodomain binds DNA elements containing a TAAT core motif [31, 32]. Four putative Dlx-2 binding sites were found in the GLS1 promoter (Figure 1E). The ChIP assay showed that Dlx-2 binds to the GLS1 promoter (Figure 1E), suggesting that Dlx-2 may directly induce GLS1 expression.

Because Dlx-2 is induced by TGF-β and Wnt [34, 37], we further investigated whether TGF-β and Wnt3a induced GLS1 expression. TGF-β and Wnt3a induced GLS1 expression (Figure 1F–1O). shDlx-2, but not shSnail, decreased TGF-β- and Wnt3a-induced GLS1 expression (Figure 1F, 1G, 1K and 1L), indicating that TGF-β and Wnt3a induce GLS1 expression in a Dlx-2 dependent and Snail-independent manner. A ChIP assay showed that TGF-β and Wnt3a increased Dlx-2 binding at the GLS1 promoter (Figure 1H and 1M). TGF-β-induced GLS1 expression was also prevented by knockdown of Smad components of the TGF-β signaling pathway (Figure 1I and 1J). shβ-catenin, shTCF4, and shAxin1/2 also suppressed Wnt3a-induced GLS1 expression (Figure 1N and 1O).

### shGLS1 inhibits *in vivo* tumor growth and metastasis

**Figure 2:**
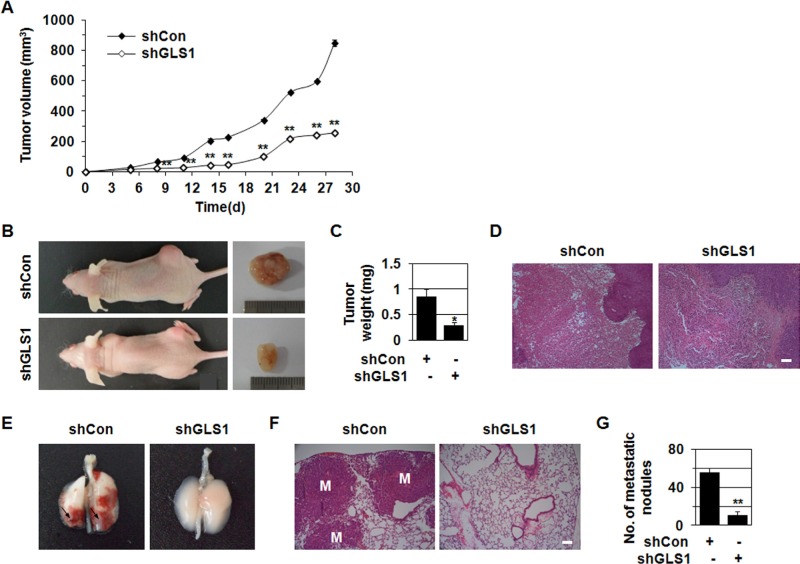
shGLS1 inhibits tumor growth and metastasis **A-D.** HCT116 cells stably transfected with shCon or shGLS1 were injected subcutaneously into the dorsal flank of nude mice (*n* = 4-5). Tumor growth curves (A) and photographs of representative mice, tumors (B), tumor weight (C) and H&E staining (D) are shown. *p < 0.05; **p < 0.01 versus shCon. **E-G.** 1 × 10^6^ HCT116 cells stably transfected with shCon or shGLS1 were injected into the tail vein of nude mice (*n* = 3-5). Photographs of representative lungs (E) and H&E staining of lung sections are shown (F). The number of metastatic nodules (G). **p < 0.01 versus shCon. All error bars represent SE. All scale bars represent 100 μm.

Because shGLS1 and GLS inhibitors reduce growth in several types of cancer cell xenografts [10, 14, 17], we examined the *in vivo* effects of shGLS1 on tumor growth and metastasis. HCT116 cells stably transfected with shCon or shGLS1 were injected subcutaneously into the dorsal flank of nude mice, which were then monitored for tumor growth. shGLS1 reduced tumor growth compared to shCon throughout the experiment (Figure 2A–2D). 28 days after injection, shGLS1 decreased tumor volume by 30% and weight by 64% relative to shCon (Figure 2A–2C).

Metastasis is the most common cause of death in cancer patients [20, 21, 23]. Thus, we investigated the effect of shGLS1 on pulmonary metastasis *in vivo*. HCT116 cells stably transfected with shCon or shGLS1 were injected into the lateral tail vein of nude mice. 48 days after injection, lungs were harvested to evaluate tumor metastasis. shGLS1 reduced the number of metastatic nodules (Figure 2E–2G) and the number of micrometastatic lesions (Figure 2F). While shCon cell injections resulted in an average of 55 metastatic nodules per lung, shGLS1 cell injections resulted in an average of 10 nodules per lung (Figure 2G). These results show that GLS1 levels are important in *in vivo* tumor metastasis as well as tumor growth.

### Inhibition of Gln metabolism prevents EMT

**Figure 3:**
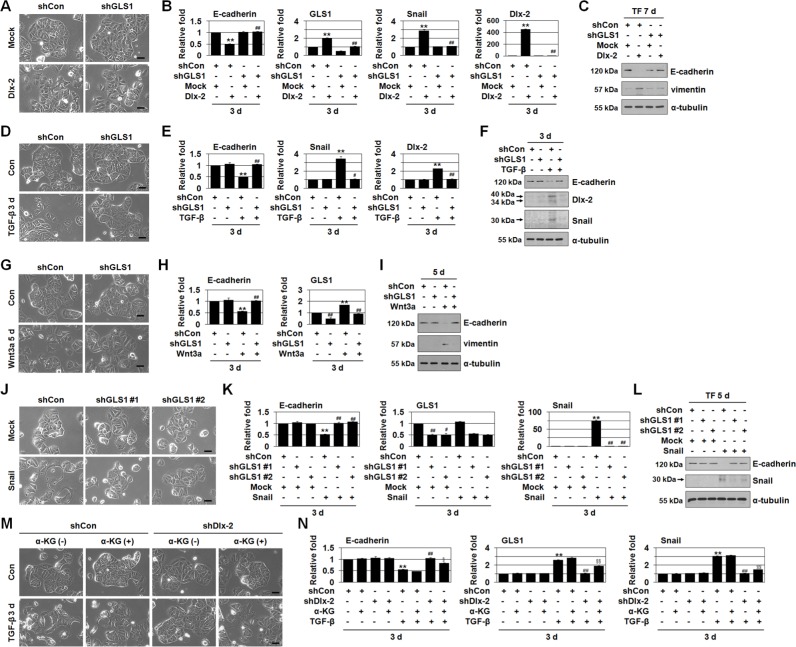
Inhibition of Gln metabolism prevents EMT **A-C.** MCF-7 cells were co-transfected with Dlx-2 and shGLS1. The cells were analyzed for cell morphology by phase-contrast microscopy (A). The cells were also analyzed by real-time qrtPCR (B) and immunoblotting (C) using the indicated primers and antibodies. **p < 0.01 versus Mock, ^##^*p* < 0.01 versus shCon. **D-I.** MCF-7 cells were transfected with shGLS1 and then treated with TGF-β (D-F) or Wnt3a CM (G-I). The cells were analyzed by phase-contrast microscopy for cell morphology (D, G), and by real-time qrtPCR (E, H) and immunoblotting (F, I) using the indicated primers and antibodies. **p < 0.01 versus untreated, ^#^*p* < 0.05; ^##^*p* < 0.01 versus shCon. **J-L.** MCF-7 cells were co-transfected with Snail and 2 different shGLS1 constructs (#1 and #2). The cells were analyzed for cell morphology by phase-contrast microscopy (J). The cells were also analyzed by real-time qrtPCR (K) and immunoblotting (L) using the indicated primers and antibodies. **p < 0.01 versus Mock, ^#^*p* < 0.05; ^##^*p* < 0.01 versus shCon. **M, N.** MCF-7 cells were transfected with shDlx-2 and then treated with TGF-β in the presence or absence of α-KG and analyzed for cell morphology by phase-contrast microscopy (M). The cells were also analyzed by real-time qrtPCR for E-cadherin, GLS1, and Snail expression (N). **p < 0.01 versus untreated, ^##^*p* < 0.01 versus shCon, ^§^*p* < 0.05; ^§§^*p* < 0.01 versus shDlx-2 in the absence of α-KG. All error bars represent SE. All scale bars represent 100 μm. For all immunoblotting images, cropped blots are shown.

EMT is closely associated with tumor metastasis [19–22]. Thus, we examined the effects of GLS1 knockdown on EMT in MCF-7 cells. shGLS1 prevented Dlx-2-induced EMT and E-cadherin downregulation (Figure 3A–3C). In addition, shGLS1 prevented TGF-β- and Wnt3a-induced EMT and E-cadherin downregulation (Figure 3D–3I). In contrast, shGLS2 did not inhibit Dlx-2-, TGF-β-, or Wnt3a-induced EMT (data not shown).

We then examined the effects of shGLS1 in Snail overexpressing cells. We used 2 different GLS1 shRNAs (shGLS1 #1 and #2), both of which effectively reduced GLS1 levels. Both shGLS1s prevented Snail-induced EMT and E-cadherin downregulation in the Snail-overexpressing MCF-7 cells (Figure 3J–3L). Thus, although GLS1 levels were not changed by Snail overexpression, Snail-induced EMT was inhibited by shGLS1.

We also examined the effects of Gln deprivation on EMT. Gln deprivation suppressed Dlx-2-induced EMT, E-cadherin downregulation, and vimentin upregulation (Supplemental Figure 1A-C). Additionally, Gln deprivation suppressed TGF-β-induced EMT and E-cadherin downregulation (Supplemental Figure 1D and 1E). Gln deprivation also suppressed Snail-induced EMT, E-cadherin downregulation, and vimentin upregulation (Supplemental Figure 1F-H). In addition, the Gln analog 6-diazo-5-oxo-L-norleucine (DON) and two GLS-selective inhibitors, compound 968 and BPTES [16], suppressed TGF-β-induced EMT and E-cadherin downregulation (Supplemental Figure 1I-N).

Gln is converted to glutamate, and then to α-ketoglutarate (α-KG), by glutamate dehydrogenase (GLUD1) [11]. Thus, we examined the effects of α-KG on reduced EMT following inhibition of Gln metabolism. We used dimethyl-α-ketoglutarate, a cell permeable α-KG precursor, to restore α-KG levels in Gln metabolism-inhibited cells. Exposing cells to α-KG prevented inhibition of EMT following Gln deprivation and DON application (Supplemental Figure 1D, 1E, 1I and 1J). Furthermore, α-KG prevented shDlx-2-mediated suppression of TGF-β-induced EMT (Figure 3M and 3N), supporting that Dlx-2 induces EMT by inducing GLS1 expression and Gln metabolism.

Gln metabolism inhibition had similar effects on EMT in HCT116 and MDCK cells. shGLS1 prevented Dlx-2-, Wnt3a-, and Snail-induced EMT and E-cadherin downregulation in HCT116 cells (Supplemental Figure 2A-F). shGLS1 also inhibited TGF-β- and Snail-induced EMT in MDCK cells (Supplemental Figure 2G-J). Thus, inhibition of Gln metabolism reduced EMT not only in MCF-7 cells, but also in HCT116 and MDCK cells.

### Gln metabolism inhibition and shDlx-2 decrease Snail mRNA levels

Because Gln metabolism inhibition prevents Dlx-2-, TGF-β-, Wnt3a-, and Snail-induced EMT, we examined the effects of Gln metabolism on Snail expression. shGLS1 or Gln deprivation reduced Snail expression in Dlx-2-overexpressing cells (Figure 3B and Supplemental Figure 1B). In addition, Gln metabolism inhibition via shGLS1, Gln deprivation, DON, 968, or BPTES also prevented TGF-β-induced Snail expression (Figure 3E and 3F, and Supplemental Figure 1E, 1J, 1L, and 1N). α-KG prevented the reduction of TGF-β-induced Snail expression following Gln metabolism inhibition (Gln deprivation or DON) (Supplemental Figure 1E and 1J).

shGLS1 or Gln deprivation also decreased Snail expression in Snail-overexpressing cells (Figure 3K and 3L, and Supplemental Figure 1G). Dlx-2 expression was also reduced by Gln metabolism inhibition (shGLS1, Gln deprivation, or DON) in Dlx-2-overexpressing cells and TGF-β-treated cells (Figure 3B, 3E, and 3F, and Supplemental Figure 1B, 1E, and 1J). Furthermore, shDlx-2 suppressed the TGF-β-mediated increase in Snail mRNA levels; α-KG prevented the inhibitory effects of shDlx-2 on TGF-β-induced Snail mRNA expression (Figure 3N).

Gln metabolism inhibition had similar effects on Snail expression in HCT116 and MDCK cells. shGLS1 prevented Dlx-2- and Snail-induced Snail expression in HCT116 cells (Supplemental Figure 2B and 2F). shGLS1 also inhibited TGF-β- and Snail-induced Snail expression in MDCK cells (Supplemental Figure 2H and 2J). Thus, Gln metabolism inhibition decreased Snail expression in HCT116 and MDCK cells in addition to MCF-7 cells.

Although co-transfection of Dlx-2 and Snail did not affect Snail expression, shDlx-2 and Snail co-transfection significantly reduced Snail levels (Supplemental Figure 3A), indicating that Dlx-2 is required for the maintenance of Snail mRNA levels. Gln metabolism inhibition and shDlx-2 together may therefore affect Snail mRNA stability. We examined this possibility by treating Snail-transfected cells with actinomycin D in the presence or absence of Gln. As expected, Snail mRNA stability was significantly reduced by Gln deprivation compared to normal growth medium (Supplemental Figure 3B). Snail mRNA had a half-life (t_1/2_) of 9.99 h in complete medium and a t_1/2_ of 3.57 h in Gln-free medium; the absence of Gln accelerated Snail mRNA decay. Thus, in addition to inducing Snail gene transcription, Dlx-2 upregulates Snail levels by stimulating Gln metabolism, which increases Snail mRNA stability.

### Gln metabolism inhibition and shDlx-2 increase the expression of Snail-targeting miRNAs through p53 regulation

Snail mRNA levels are suppressed by many miRNAs, including miR-23b, miR-29b, miR-30, miR-34, miR-125b, miR-148a, miR-153, miR-200, miR-203, miR let-7, miR-7, miR-9, miR-128-2, miR-145, and miR-204 (39; Supplemental references 1-17). We examined whether Gln metabolism inhibition affects the expression of these Snail-targeting miRNAs. shGLS1, DON, and Gln deprivation increased the expression of miR-23b, miR-29b, miR-30, miR-34, miR-125b, miR-148a, miR-153, miR-200, and miR-203 without affecting the expression of miR let-7, miR-7, miR-9, miR-128-2, miR-145, and miR-204 (Table 1). Similar results were obtained in Dlx-2 knockdown cells (Table 1). α-KG inhibited the effects of shGLS1, Gln deprivation, and shDlx-2 on the expression of the Snail-targeting miRNAs (Table 1).

**Table 1:** p53-dependent regulation of Snail-targeting miRNAs by Gln metabolism inhibition and shDlx-2

Genes	shGLS1	shGLS1		shGLS1		DON		Gln (−)	Gln (−)		Gln (−)		shDlx-2	shDlx-2		shDlx-2	
	3 d	α-KG (−)	α-KG (+)	shCon	shp53	shCon	shp53	3 d	α-KG (−)	α-KG (+)	shCon	shp53	3 d	α-KG (−)	α-KG (+)	shCon	shp53
miR-23b	2.813^aa^	2.886^aa^	1.095^bb^	2.923^aa^	1.084^bb^	2.464^aa^	1.068^bb^	2.525^aa^	2.491^aa^	0.996^b^	2.508^aa^	1.093^bb^	1.871^aa^	1.872^aa^	1.013^bb^	1.890^aa^	1.088^bb^
miR-29b	1.551^aa^	1.576^aa^	1.046^b^	1.571^aa^	1.054^bb^	2.037^a^	1.055^b^	2.021^aa^	2.071^aa^	0.997^b^	1.828^a^	1.046^b^	1.562^aa^	1.610^aa^	0.981^bb^	1.541^aa^	1.040^bb^
miR-30	2.505^aa^	2.532^aa^	0.877^bb^	2.548^aa^	1.018^bb^	2.382^a^	1.127^b^	2.465^aa^	2.493^aa^	0.954^bb^	2.414^aa^	1.058^bb^	1.848^aa^	1.823^aa^	1.003^b^	1.859^aa^	1.063^bb^
miR-34	2.156^aa^	2.135^aa^	1.004^bb^	2.175^aa^	1.020^bb^	2.623^a^	1.071^b^	2.464^aa^	2.448^aa^	0.937^b^	2.365^aa^	1.039^bb^	1.951^aa^	1.835^a^	0.914^bb^	1.918^aa^	1.003^bb^
miR-125b	3.544^aa^	3.570^a^	1.024^b^	3.656^aa^	1.081^bb^	3.185^a^	1.045^b^	3.215^aa^	3.078^aa^	1.051^bb^	3.324^aa^	1.053^bb^	2.983^aa^	3.045^aa^	1.052^bb^	2.880^aa^	1.101^bb^
miR-148a	3.139^aa^	3.267^aa^	0.917^b^	3.048^aa^	1.043^bb^	2.874^a^	1.073^b^	3.003^aa^	3.052^aa^	1.023^bb^	2.919^aa^	1.062^bb^	2.356^aa^	2.438^aa^	1.018^bb^	2.239^aa^	1.007^bb^
miR-153	3.124^aa^	3.130^aa^	1.150^bb^	3.218^aa^	1.080^bb^	2.458^a^	1.092^b^	2.577^aa^	2.501^aa^	1.178^b^	2.770^aa^	1.108^bb^	2.472^aa^	2.599^aa^	0.981^bb^	2.344^aa^	1.043^bb^
miR-200	2.616^aa^	2.700^aa^	0.930^bb^	2.618^aa^	1.067^bb^	2.261^a^	1.070^b^	2.472^aa^	2.471^aa^	1.032^bb^	2.480^aa^	1.082^bb^	2.183^aa^	2.318^aa^	0.993^bb^	2.123^aa^	1.074^bb^
miR-203	2.257^aa^	2.363^aa^	1.167^bb^	2.289^aa^	1.073^bb^	2.196^a^	1.083^b^	2.376^aa^	2.312^aa^	1.078^bb^	2.380^aa^	1.037^bb^	2.271^aa^	2.306^aa^	1.038^bb^	2.223^aa^	1.044^bb^
miR let-7	1.001							1.075	1.016	1.107			0.964				
miR-7	1.230							1.264	1.306	1.386			1.206				
miR-9	0.917							0.899	0.846	0.866			1.064				
miR-128-2	1.275							1.119	1.022	0.981			1.061				
miR-145	0.763							1.051	1.106	0.929			1.066				
miR-204	1.102							1.123	1.069	1.103			1.020				

GLS1, glutaminase; DON, 6-diazo-5-oxo-L-norleucine; Gln, glutamine; Dlx-2, distal-less (Dlx) homeobox 2; α-KG, dimethyl-α-ketoglutarate; miR, microRNA.

MCF-7 cells were transfected with shGLS1, cultured in complete or Gln-free medium or transfected with shDlx-2, and then treated with α-KG or co-transfected with shp53. MCF-7 cells were transfected with shp53 and then treated with DON; The cells were analyzed by real-time qrtPCR.

a *p* < 0.05

aa *p* < 0.01 versus control (untreated and shCon).

b *p* < 0.05

bb *p* < 0.01 versus shGLS1, DON, Gln-free medium, or shDlx-2.

We also found that TGF-β reduced the expression of the Snail-targeting miRNAs (Supplemental Figure 4A). These results suggest that TGF-β upregulates Snail levels by reducing the expression of Snail-targeting miRNAs in addition to directly increasing Snail gene expression. Gln deprivation suppressed the inhibitory effects of TGF-β on Snail-targeting miRNA levels (Supplemental Figure 4A). In addition, Dlx-2 overexpression reduced the expression of the Snail-targeting miRNAs (Supplemental Figure 4B). Thus, Dlx-2 increased Snail levels by repressing Snail-targeting miRNA expression; furthermore, Dlx-2 may downregulate Snail-targeting miRNAs through regulation of Gln metabolism.

**Figure 4:**
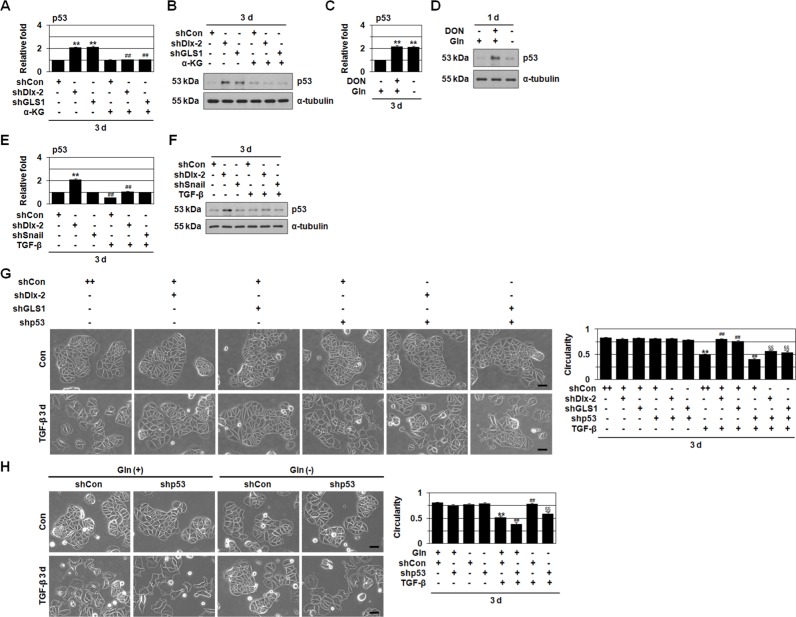
shDlx-2 or Gln metabolism inhibition increase the expression of p53 **A, B.** MCF-7 cells transfected with shDlx-2 or shGLS1 and then treated with α-KG were analyzed by real-time qrtPCR (A) and immunoblotting (B) for p53 expression. **p < 0.01 versus shCon, ^##^*p* < 0.01 versus untreated. **C, D.** MCF-7 cells treated with DON or cultured in Gln-free medium were analyzed by real-time qrtPCR (C) and immunoblotting (D) for p53 expression. **p < 0.01 versus control. **E, F.** MCF-7 cells transfected with shDlx-2 or shSnail and then treated with TGF-β were analyzed by real-time qrtPCR (E) and immunoblotting (F) for p53 expression. **p < 0.01 versus shCon, ^##^*p* < 0.01 versus untreated. **G.** MCF-7 cells co-transfected with shDlx-2 or shGLS1 and shp53 and then treated with TGF-β were analyzed by phase-contrast microscopy for cell morphology (left) and circularity (right). The borders (white) drawn along the cell edges are shown for quantification of circularity. The results (54-158 cells in each group) are presented as mean ± SE. **p < 0.01 versus untreated, ^##^*p* < 0.01 versus shCon with TGF-β, ^§§^*p* < 0.01 versus shDlx-2 or shGLS1 with TGF-β. **H.** MCF-7 cells transfected with shp53 and then cultured in complete or Gln-free medium with TGF-β were analyzed by phase-contrast microscopy for cell morphology (left) and circularity (right). The borders (white) drawn along the cell edges are shown for quantification of circularity. The results (26-134 cells in each group) are presented as mean ± SE. **p < 0.01 versus untreated, ^##^*p* < 0.01 versus complete medium with TGF-β, ^§§^*p* < 0.01 versus shCon in Gln-free medium with TGF-β. All error bars represent SE. All scale bars represent 100 μm. For all immunoblotting images, cropped blots are shown.

Tumor cells exhibit enhanced Glc and Gln uptake and are especially sensitive to nutritional stress. The tumor suppressor p53, which reprograms tumor cell functions in response to nutritional stress, is a major sensor of low nutrient levels [40]. Therefore, we investigated whether p53 is involved in the expression of Snail-targeting miRNAs. Gln metabolism inhibition (shGLS1, DON, or Gln deprivation) and shDlx-2 increased p53 expression (Figure 4A–4D), while α-KG suppressed shGLS1- and shDlx-2 induced p53 expression (Figure 4A and 4B). TGF-β decreases p53 by inhibiting its transcription and translation [41]. We found that shDlx-2 prevented the TGF-β-mediated decrease in p53 levels, but shSnail did not (Figure 4E and 4F). This indicates that TGF-β decreases p53 via Dlx-2. The shDlx-2-mediated increase in p53 levels was also prevented by TGF-β (Figure 4E and 4F).

In addition, shp53 suppressed the expression of all of 9 Snail-targeting miRNAs induced by Gln metabolism inhibition (shGLS1, DON, and Gln deprivation) and shDlx-2 (Table 1). Thus, p53 induces the expression of other miRNAs besides miR-34 and miR-200 during Gln metabolism inhibition.

We then examined the role of p53 in shDlx-2- and Gln metabolism inhibition-mediated suppression of TGF-β-induced EMT. shp53 promoted TGF-β-induced EMT (Figure 4G and 4H). shp53 prevented shDlx-2- and shGLS1-mediated suppression of TGF-β-induced EMT (Figure 4G). shp53 prevented Gln deprivation-mediated suppression of TGF-β-induced EMT in MCF-7 cells as well (Figure 4H), suggesting that TGF-β induces EMT in a p53-dependent manner. In summary, Dlx-2 and Gln metabolism may downregulate Snail-targeting miRNAs in a p53-dependent manner during TGF-β-induced EMT.

### Inhibition of Gln metabolism prevents Dlx-2/TGF-β/Wnt3a/Snail-induced glycolytic switch and mitochondrial repression

**Figure 5:**
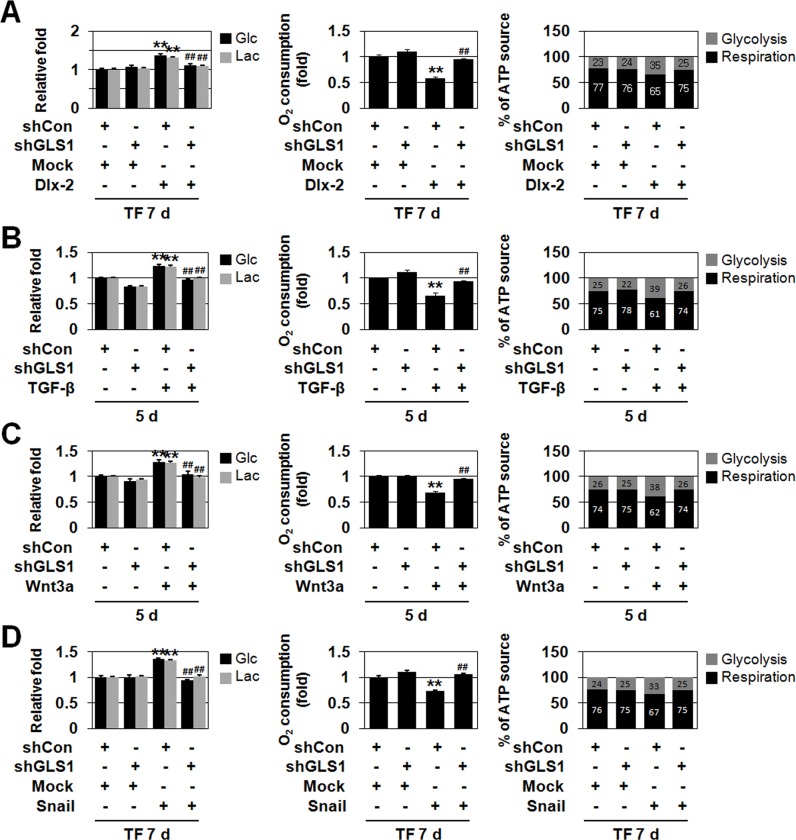
Gln metabolism is linked to TGF-β-, Wnt3a-, and Dlx-2/Snail-induced glycolytic switch and mitochondrial repression **A.** MCF-7 cells were co-transfected with Dlx-2 and shGLS1. The cells were analyzed for Glc consumption, Lac production, mitochondrial respiration, and ATP source. **p < 0.01 versus Mock, ^##^*p* < 0.01 versus shCon. **B, C.** MCF-7 cells were transfected with shGLS1 and then treated with TGF-β (B) or Wnt3a CM (C). The cells were analyzed for Glc consumption, Lac production, mitochondrial respiration, and ATP source. **p < 0.01 versus untreated, ^##^*p* < 0.01 versus shCon. **D.** MCF-7 cells were co-transfected with Snail and shGLS1. The cells were analyzed for Glc consumption, Lac production, mitochondrial respiration and ATP source. **p < 0.01 versus Mock, ^##^*p* < 0.01 versus shCon. The amount of ATP produced by aerobic respiration (black bars) and glycolysis (gray bars) was calculated by measuring oxygen consumption and Lac production in the cells (right panels in A-D). All error bars represent SE.

Previously, we showed that Snail induces glycolytic switch and mitochondrial repression [29]. Dlx-2 induced glycolytic switch and mitochondrial repression (Figure 5A and Supplemental Figure 5A). Next, we examined whether Gln metabolism affects Dlx-2-induced glycolytic switch and mitochondrial repression. shGLS1 and Gln deprivation suppressed Dlx-2-induced Glc consumption and Lac production (Figure 5A and Supplemental Figure 5A). In addition, shGLS1 and Gln deprivation prevented Dlx-2-induced repression of oxygen (O_2_) consumption (Figure 5A and Supplemental Figure 5A). ATP levels in these cells were similar to those in control cells (data not shown). By measuring O_2_ consumption and Lac production, we estimated the relative contributions of glycolysis and aerobic respiration to total ATP production. shGLS1 and Gln deprivation prevented the Dlx-2-mediated increase in the ratio of ATP produced by glycolysis versus aerobic respiration (Figure 5A and Supplemental Figure 5A). This indicates that Gln metabolism inhibition suppresses Dlx-2-induced glycolytic switch and mitochondrial repression.

We then examined whether Gln metabolism is linked to TGF-β/Wnt-induced glycolytic switch and mitochondrial repression. TGF-β and Wnt induced glycolytic switch and mitochondrial repression (Figure 5B and 5C). shGLS1 suppressed TGF-β- and Wnt-induced Glc consumption and Lac production (Figure 5B and 5C). In addition, shGLS1 prevented TGF-β- and Wnt-induced repression of O_2_ consumption (Figure 5B and 5C). Total ATP concentrations remained the same in all cells (data not shown). shGLS1 inhibited the TGF-β- and Wnt-mediated increase in the ratio of ATP produced by glycolysis versus aerobic respiration (Figure 5B and 5C). This indicates that shGLS1 suppresses TGF-β/Wnt-induced glycolytic switch and mitochondrial repression. Thus, Gln metabolism appears to be linked to Dlx-2-, TGF-β-, and Wnt-induced glycolytic switch and mitochondrial repression.

Finally, we examined whether Gln metabolism affects Snail-induced glycolytic switch and mitochondrial repression (Figure 5D and Supplemental Figure 5B). Similar to their inhibitory effects on EMT, shGLS1 and Gln deprivation prevented Snail-induced glycolytic switch in Snail-overexpressing cells (Figure 5D and Supplemental Figure 5B). shGLS1 and Gln deprivation also prevented Snail-induced repression of O_2_ consumption (Figure 5D and Supplemental Figure 5B). Thus, Gln metabolism inhibition suppressed Snail-induced glycolytic switch and mitochondrial repression.

### Expression of Dlx-2, GLS1, Snail, p53, and Snail-targeting miRNAs in human tumors

**Figure 6:**
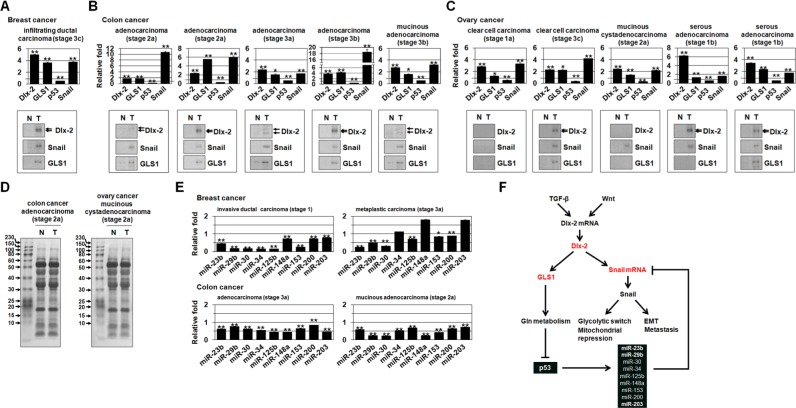
The expression of Dlx-2, GLS1, p53, Snail, and Snail-targeting miRNAs in human tumors **A-C.** Real-time qrtPCR data showing expression of Dlx-2, GLS1, p53 and Snail mRNA, and immunoblotting for Dlx-2, Snail, and GLS1 protein in normal (N) and tumor (T) tissues from the indicated tumor types and histological stages (TNM classification) of breast (A), colon (B) and ovarian cancer (C). **D.** Ponceau S-stained membranes showing loading of same amount of proteins. **E.** Real-time qrtPCR data of Snail-targeting miRNAs in normal (N) and tumor (T) tissues in the indicated tumor types. Relative levels of miRNAs were normalized to the corresponding normal tissues. *p < 0.05; **p < 0.01 versus matched normal (N) tissues. All error bars represent SE. **F.** A schematic representation showing a new function of Dlx-2/GLS1-driven Gln metabolism that contributes to Snail-dependent EMT and glycolytic switch. For all immunoblotting images, cropped blots are shown.

To examine the physiological relevance of the Dlx-2/GLS1/p53/miRNA/Snail cascade, we analyzed their levels in human tumor samples. Dlx-2, GLS1, Snail, p53, and Snail-targeting miRNA expression was examined with real-time qrtPCR using RNAs extracted from paired biopsies of breast, colon, and ovarian cancer and corresponding normal tissues. Dlx-2 and Snail expression were higher in breast cancer tissues compared to matched non-tumorigenic tissues (Figure 6A). GLS1 expression was also higher, and p53 expression was lower, in breast cancer tissues (Figure 6A). In addition, Dlx-2, GLS1, and Snail expression were higher, and p53 expression was lower, in colon and ovarian cancer tissues compared to matched normal tissues regardless of cancer stage (Figure 6B–6D). The expression of most Snail-targeting miRNAs (miR-23b, miR-29b, miR-30, miR-125b, miR-153, and miR-200) was lower in breast and colon cancer than in matched normal tissues; however, miR-34, miR-148a, and miR-203 expression was similar to control tissues in metaplastic breast carcinoma (Figure 6E). We also examined the expression of Dlx-2, GLS1, and Snail protein using immunoblotting. The expression of all three was higher in breast, colon and ovarian cancer tissues than in matched non-tumorigenic tissues (Figure 6A–6D). These results support important roles for Dlx-2, GLS1, and p53 in tumor development.

## DISCUSSION

Dlx-2, a transcriptional factor involved in embryonic development, tissue homeostasis, and the cell cycle [31, 32], is important in carcinogenesis; Dlx-2 expression correlates positively with more advanced cancer stages and with poor prognosis in a variety of human cancer types [33–36]. In this study, we show that Dlx-2 induces EMT and regulates GLS1 expression and Gln metabolism, which increases tumor metastasis. The Dlx-2 target gene GLS1 (Figure 1) is important in Gln metabolism. GLS1 is a critical enzyme in a number of cancers [12–17]. GLS1 levels are increased in breast and prostate cancers and HCC tissues compared to matched normal tissues and positively correlate with both degree of malignancy and tumor grade [17, 18] (Figure 6). c-Myc-mediated repression of miR-23a/b increases GLS1 levels [12, 13]. In addition, the NF-κB member p65 subunit suppresses the expression of miR-23a and directly inhibits GLS1 expression [42]. Xiang *et al*. (2015) showed that *GLS1* mRNA is upregulated in primary human HCC compared to surrounding non-tumor tissue, and that, while *MYC* mRNA levels were also elevated in the tumors, they did not correlate directly with *GLS1* mRNA levels [17]. This suggests that factors other than MYC may be involved in the upregulation of *GLS1* mRNA levels in HCC. Here, GLS1 was induced by Dlx-2 (Figure 1). We showed that GLS1 expression was also upregulated by TGF-β and Wnt in a Dlx-2-dependent, but Snail-independent, manner (Figure 1). Furthermore, Dlx-2 bound to the GLS1 promoter (Figure 1E), indicating that Dlx-2 induces GLS1 expression. Whether Dlx-2 binding is required for Dlx-2-induced GLS1 activation remains to be elucidated through the use of transcriptional reporter assays combined with site-directed mutagenesis.

We showed that shGLS1 inhibits tumor growth and metastasis *in vivo* (Figure 2). shGLS1 and GLS-specific inhibitors, BPTES and 968, reduce the growth of several types of cancer cell xenografts [10, 14, 17]. However, the effects of shGLS1 and GLS1 inhibitors on metastasis have been not reported. Our results show an important role for the Dlx-2-GLS1/Gln metabolism cascade in tumor metastasis as well as in tumor growth.

Because EMT is an essential process in the initiation of metastasis [19–23], we investigated the effects of GLS1/Gln metabolism inhibition on EMT. Gln metabolism inhibition by shGLS1, Gln deprivation, and Gln metabolism inhibitors (DON, 968, BPTES) prevented Dlx-2-, TGF-β-, and Wnt-induced EMT, indicating that GLS1/Gln metabolism may be involved in Dlx-2/TGF-β/Wnt-induced EMT (Figure 3, and Supplemental Figure 1 and 2). The Dlx-2-GLS1-Gln metabolism axis is thought to be an essential regulator of TGF-β- and Wnt-induced EMT. Interestingly, shGLS1 also inhibited Snail-induced EMT, although GLS1 expression was not induced by Snail (Figure 3J–3L). Thus, the Dlx-2-GLS1-Gln axis may provide metabolic support for Snail-induced EMT, rather than acting as an upstream pathway.

Gln metabolism inhibition together with shGLS1 or Gln deprivation prevented Dlx-2-, TGF-β-, Wnt-, and Snail-induced glycolytic switch, indicating that Gln metabolism is closely linked to glycolytic switch (Figure 5, and Supplemental Figure 5). Recently, long-term exposure of cancer cells to acidic pH (resulting from glycolysis) has been shown to upregulate Gln metabolism to ensure intracellular pH homeostasis [43]. Thus, Gln and Glc metabolism influence each other in cancer cells.

Gln metabolism inhibition decreased Snail mRNA stability (Figure 3, and Supplemental Figure 3B). In addition, shDlx-2, shGLS1, Gln deprivation, and DON increased the expression of several Snail-targeting miRNAs (Table 1). Thus, the Dlx-2/GLS1/Gln metabolism pathway seems to increase Snail mRNA stability by regulating Snail-targeting miRNAs (Figure 6F).

We further showed that Gln metabolism inhibition increased levels of p53 protein and mRNA (Figure 4A–4D). Recently, it was shown that Gln deprivation induced p53 through B55α [44]. Here, shDlx-2, but not shSnail, increased p53 expression (Figure 4A, 4B, 4E and 4F). In addition, α-KG suppressed shDlx-2- and shGLS1-induced p53 expression (Figure 4A and 4B), indicating that p53 is downregulated by Dlx-2-driven Gln metabolism. Snail-targeting miRNA expression was upregulated by p53 (Table 1). Our results indicate that Snail mRNA stability is linked to p53 levels. Taken together, Gln metabolism inhibition prevented TGF-β-induced EMT and glycolytic switch by increasing Snail-targeting miRNA expression via p53, ultimately decreasing Snail stability (Figure 6F).

p53 is the strongest tumor suppressor gene, and almost all human cancers involve the loss of p53 function [45]. Dlx-2-induced Gln metabolism likely contributes to p53 reduction during tumor development. Gln deprivation induced p53 via ROS-dependent B55α activation [44]. Thus, Gln metabolism inhibition may re-activate p53. In addition, TGF-β-induced EMT was more prominent in shp53-treated MCF-7 cells than in shCon-treated MCF-7 cells (Figure 4G and 4H). p53 activates the transcription of miR-200 and miR-34 family members [39, 46, 47]. Furthermore, loss of p53 function and p53 mutations promote cancer cell EMT by decreasing miRNA-34 levels in colon, breast, and lung carcinoma cells, which disinhibits Snail protein expression and activity [39].

We found that Gln metabolism inhibition also prevented Wnt3a-induced EMT and glycolytic switch (Figure 3G–3I and 5C, and Supplemental Figure 2C and 2D). TGF-β induces Snail mRNA levels though Dlx-2 activation. However, Wnt increased Snail levels in a Dlx-2-independent manner. Although the mechanisms by which TGF-β and Wnt3a induce Snail expression differ, the effects of Gln metabolism inhibition on Wnt3a-induced EMT and glycolytic switch were similar. Wnt signaling induces EMT by activating an Axin2 pathway that stabilizes Snail. Axin2 stimulates EMT by acting as a chaperone for nuclear export of GSK3β, the dominant kinase responsible for Snail protein turnover [48]. miR-34 directly suppresses Axin2 and other Wnt signaling molecules, including β-catenin and LEF1, in addition to Snail, ultimately suppressing EMT [39, 49]. Therefore, Gln metabolism inhibition seems to prevent Wnt3a-induced EMT and glycolytic switch through p53-dependent expression of miR-34.

We further showed that Dlx-2 levels were also reduced by Gln metabolism inhibition (shGLS1, Gln deprivation or DON) in Dlx-2-overexpressing cells and TGF-β-treated cells (Figure 3B, 3E and 3F, and Supplemental Figure 1B, 1E, 1J, 2B and 2H). We identified Snail-targeting miRNAs that might bind to the 3′-UTRs of Dlx-2 using miRanda, a target prediction program. Several miRNAs, including miR-23b and miR-203, were predicted to bind to the Dlx-2 3′-UTR (data not shown).

Our results showed that the Dlx-2/GLS1/Gln metabolism axis is involved in TGF-β/Wnt/Snail-induced EMT and glycolytic switch. Dlx-2 increased Snail mRNA stability by activating GLS1/Gln metabolism, which inhibited p53-dependent upregulation of Snail-targeting miRNAs (Figure 6F). Dlx-2 induces Snail gene expression [37]. Thus, Dlx-2 may induce Snail mRNA expression through two mechanisms; (i) activation of Snail mRNA transcription at an early time point, and (ii) increased Snail mRNA stability at a late time point.

Gln deprivation prevented Dlx-2-, TGF-β-, Wnt-, and Snail-induced EMT by reducing Snail mRNA levels through p53-dependent upregulation of Snail-targeting miRNAs. Gln activates the highly conserved kinase mammalian target of rapamycin complex 1 (mTORC1), which stimulates protein translation and cell growth; thus, Gln deprivation strongly suppresses growth by inhibiting mTORC1 in several types of cells [50–52]. Gln deprivation also inhibits global protein translation by reducing GCN2 protein kinase-mediated phosphorylation of the translation initiation factor eIF2α [53, 54]. Many studies have shown that mTOR signaling activates EMT, cancer invasion, and metastasis [55–57]. Although genetic and pharmacologic inhibition of mTORC1 triggers EMT in normal immortalized human epithelial cell lines and primary epithelial cells [58], mTORC1 inhibition represses EMT in colon and breast cancer cells [59]. This difference in the effect of mTORC1 inhibition on EMT is likely due to differences in the mutational status of the cells [59]. Cai *et al*. (2014) showed that mTORC1 induces phosphorylation of the cap-dependent translation repressor 4E-BP1, which leads to its dissociation from eIF4E and formation of the translation initiation complex. This results in cap-dependent activation of Snail translation and the induction of EMT in cancer cells [59]. mTORC1/4E-BP1-mediated Snail translation may also contribute to Dlx-2/Gln metabolism-induced Snail expression. Whether Dlx-2/GLS1/Gln metabolism activates mTORC1 signaling requires further investigation.

Oncogenic metabolism, including Gln metabolism, has been suggested to confer growth advantages to cancer cells by providing biosynthetic precursors [2, 4–8]. We propose that, in addition to its well-described role in supporting tumor growth by providing metabolites, Gln metabolism may also contribute to tumor EMT, metastasis, and progression. Metastasis is a complex process and includes detachment of tumor cells from the primary site. The loss of cell-matrix interactions induces anoikis, and resistance to anoikis is a prerequisite for tumor metastasis [60, 61]. Snail induces a glycolytic switch and suppresses mitochondrial oxidative metabolism [30], which may contribute to anoikis resistance and metastasis by preventing excess ROS generation. Thus, the Dlx-2/GLS1 cascade may promote tumor metastasis by increasing Snail-mediated anoikis resistance in addition to EMT. Our results suggest that GLS1 may be a potential therapeutic target for the prevention of metastasis and tumor progression.

## MATERIALS AND METHODS

### Cell culture

MCF-7, Madin Darby Canine Kidney (MDCK), and L cells from the American Type Culture Collection (ATCC) were cultured under established conditions [29]. Wnt3a-secreting L cells and HCT116 cells were provided by Dr. Min DS and Dr. Kim YJ (Pusan National University, Pusan, Korea), respectively. The Gln deprivation experiment was performed by applying EMEM without Gln (GIBCO, Carlsbad, CA, USA). Recombinant TGF-β (R&D Systems, MN, USA) was applied to cells at a concentration of 10 ng/ml. 40 μM DON (Sigma, St. Louis, MO, USA), 5 μM 968 (Calbiochem, San Diego, CA, USA), 10 μM BPTES (Sigma), or 1 mM dimethyl-α-ketoglutarate (α-KG; Sigma) were also applied to cells.

### Transfection and short hairpin RNA (shRNA) interference

The expression vectors pCAGGS-Dlx-2 (provided by Dr. John L.R. Rubenstein, University of California at San Francisco) and pCR3.1-Snail-Flg (provided by J.I. Yook, Yonsei University, Korea) were transfected into MCF-7, MDCK, and HCT116 cells using jetPEI (Polyplus transfection, SA, USA). pSUPER vectors for shRNA against control, Dlx-2, Snail, GLS1, Smad2, Smad3, Smad4, β-catenin, TCF4, Axin1, Axin2, and p53 (abbreviations; shCon, shDlx-2, etc.) were produced and transfected as described previously [29]. shRNA target sequences are listed in Supplementary Table S1.

### Immunoblotting, real-time qrtPCR, immunofluorescence (IF) staining, and chromatin immunoprecipitation (ChIP) assay

Immunoblotting, real-time qrtPCR, IF and ChIP assay were performed as described previously [29, 33].

### Assays for mitochondrial respiration, Glc consumption, Lac production, and ATP production

Mitochondrial respiration was measured as described previously [29, 62]. Glc, Lac and intracellular ATP levels in the media were determined using a Glc oxidation assay kit (Sigma, MO, USA), a colorimetric and fluorescence-based Lac assay kit (BioVision, California, USA), and an ATP Bioluminescence Assay kit (Roche, Switzerland), respectively, according to the manufacturers' instructions. Levels of Glc, Lac, and intracellular ATP were normalized to protein concentrations. Levels of ATP produced by aerobic respiration and glycolysis were determined by measuring Lac production and oxygen consumption [29, 63].

## SUPPLEMENTARY DATA FIGURES AND TABLES


