# Supplementary material for: Dlx-2 and glutaminase upregulate epithelial-mesenchymal transition and glycolytic switch
Source: Oncotarget. 2016 Jan 11;7(7):7925–39. doi: 10.18632/oncotarget.6879 (PMC4884964; doi:10.18632/oncotarget.6879)
Supplement: Supplementary file 1 [file oncotarget-07-7925-s001.pdf]

SUPPLEMENTARY DATA

SUPPLEMENTARY MATERIALS AND METHODS

Cell culture

MCF-7 and MDCK cells were cultured in Eagle's minimal essential medium (EMEM; Hyclone, Logan, UT, USA). L cells and Wnt3a-secreting L cells were cultured in Dulbecco's Modified Eagle Medium (DMEM; Hyclone, Logan, UT, USA). HCT116 cells were cultured in RPMI supplemented with 10% (v/v) heat-inactivated fetal bovine serum (FBS, Hyclone, Logan, UT, USA) and 1% penicillin-streptomycin (PS, Hyclone, Logan, UT, USA). All cells were cultured in a 37°C humidified incubator with 5% CO_2. Cells were routinely confirmed to be negative for mycoplasma using the Mycoplasma PCR Detection Kit (iNtRON Biotechnology).

Immunoblotting and real-time quantitative reverse transcription PCR (real-time qrtPCR)

Immunoblotting was performed with the following antibodies: Dlx-2 (Millipore, Billerca, MA, USA); Snail (Abgent, San Diego, CA, USA); GLS1 (ProteinTech Group, Chicago, IL, USA); E-cadherin, p53 and vimentin (Santa Cruz, CA, USA); α-tubulin (Biogenex, CA, USA). Total mRNA including miRNA was isolated from cells using TRIzol (Invitrogen, Carlsbad, CA, USA) according to the supplier's instructions. Transcript levels were assessed with real-time qrtPCR using primers for Dlx-2, Snail, GLS1, E-cadherin, p53 and β-actin. Values are normalized to β-actin (Supplementary Table S2). Analysis of hsa-miRNA was performed using the miScript system (QIAGEN, Milan Italy), which consists of a miScript Reverse Transcription kit, miScript Primer assays, and a miScript SYBR Green PCR kit, according to the protocol provided by the company. The reverse transcription reaction was performed starting with 2 μg of total RNA and using the miScript Reverse Transcription kit according to the manufacturer's protocol. The expression of Snail-targeting miRNA (mature hsa-miR-23b, hsa-miR-29b, hsa-miR-30, hsa-miR-34, hsa-miR-125b, hsa-miR-148a, hsa-miR-153, hsa-miR-200, hsa-miR-203, hsa-miR let-7, hsa-miR-7, hsa-miR-9, hsa-miR-128-2, hsa-miR-145, and hsa-miR-204) (Kim et al., 2011; Supplemental references 1-17), and RNU6 RNA, as a housekeeping gene, was assayed using the miScript SYBR Green PCR kit.

Immunofluorescence (IF) staining

Cells were fixed with 3.7% formaldehyde, permeabilized in 0.2% Triton X-100, and blocked with 2% BSA. The cells were then incubated overnight with anti-E-cadherin antibody and immunostained with AlexaFluor 488-conjugated goat anti-mouse secondary antibody (Invitrogen). Hoechst 33342 (Invitrogen) was used to stain cell nuclei. The cells were viewed under a fluorescence microscope.

Chromatin immunoprecipitation (ChIP) assay

ChIP assay was performed using a ChIP assay kit (Millipore) according to the manufacturer's instructions. Isotype control IgG and anti-Dlx-2 (Santa Cruz, CA, USA) were used to immunoprecipitate DNA-containing complexes. ChIP-enriched DNA was analyzed by PCR using primers complementary to the specific sequences (#1, #2 and #3) containing putative Dlx-2 binding sites at the *GLS1* promoter. Putative Dlx-2 binding sites in *GLS1* promoter region and primers are listed in Supplementary Table S2 and S3.

Animal studies

6-week-old female BALB/c nude mice were obtained from Orient (Seoul, Korea) and maintained under a 12 h light/dark cycle and in accordance with the regulations of Pusan National University. For the tumor growth assay, 2×10^6 HCT116 cells stably transfected with shCon or shGLS1 were resuspended in 100 μl of a 1:1 mixture of PBS and Matrigel (Corning, NY, USA) and injected subcutaneously into the dorsal flank of each mouse ($n = 4$-5 per group). Tumor volumes were measured 2-3 times per week with a caliper and were calculated using the formula 1/2(length × width^2). Tumor weight was determined at the endpoint. Tumors were excised, fixed in 10% formalin solution (Sigma), and embedded in paraffin for histopathological examination. For the lung-metastasis assay, 1×10^6 HCT116 cells stably transfected with shCon or shGLS1 were resuspended in 100 μl of PBS and injected into the lateral tail vein of each mouse (n = 3-5 per group). 48 days after injection, when mice had not died but some appeared to be sick, all mice were killed and their lungs were removed and fixed in 10% formalin solution. The number of surface metastatic nodules per lung was determined under a dissecting microscope. The excised lungs were embedded in paraffin. Tissue sections (5 μm) were prepared and stained with haemotoxylin and eosin (H&E). All animal protocols were approved by the Institutional Animal Care and Use Committee of Pusan National University.

Assays for mitochondrial respiration

For mitochondrial respiration assay, exponentially growing cells (1.5×10^6) were washed with TD buffer (137 mM NaCl, 5 mM KCl, 0.7 mM Na_2HPO_4, 25 mM Tris-HCl, pH 7.4), were collected, and resuspended in complete medium without phenol red. 5×10^5 cells were transferred to the Mitocell chamber equipped with a Clark oxygen electrode (782 Oxygen Meter, Strathkelvin Instruments, Glasgow, UK). Oxygen consumption rates were measured after adding 30 µM DNP to obtain maximum respiration rate, and specificity for mitochondrial respiration was confirmed by adding 5 mM KCN (Yoon et al., 2005).

Human tumor samples

All human tissues from patient #70648 (infiltrating ductal carcinoma) with breast cancer, patients #71335, #71593, #70852, #71304 (adenocarcinoma) and #70825 (mucinous adenocarcinoma) with colon cancer, and patients #1963, #1844 (clear cell carcinoma), #1903 (mucinous cystadenocarcinoma), #2281 and #2297 (serous adenocarcinoma) with ovarian cancer, and normal matched tissue pairs from the same individuals, were provided by the National Biobank of Korea, PNUH in compliance with all regulations related to biomedical research with human samples, including informed consent of the patients for the use of their samples. We performed real-time qrtPCR and immunoblotting with cancer tissues. TRIzol extraction of total RNA and subsequent extraction of protein was carried out according to the manufacturer's specifications (Invitrogen Corp.). To a 50-100 mg tissue sample, 1 ml of TRIzol was added, and the sample was homogenized for 2-3 min with a tissuelyser (QIAGEN, Hilden, Germany) at 30 Hz.

Microarray

Dlx-2 overexpressing MCF-7 cells were analyzed by microarray. Hybridization to microarrays was performed to screen for differentially expressed genes using Agilent Human Genome 8x60K array (Agilent technologies, CA); a complete listing of the genes on this microarray is available at the following web site: http://www.agilent.com. Gene expression levels were calculated with Feature Extraction v10.7.3.1 (Agilent technologies, CA). Relative signal intensities for each gene were generated using the Robust Multi-Array Average algorithm. The data were processed based on the quantile normalization method using GeneSpring GX 11.5.1 (Agilent technologies, CA). The normalized and log transformed intensity values were then analyzed using GeneSpring GX 11.5.1.

Measurement of Snail mRNA stability

MCF-7 cells were transfected with Snail for 18 h, rinsed 3 times, and then cultured in complete or Gln-free medium for the chase period with 5 µg/ml Actinomycin D (Sigma). The cells were harvested at different time points (0.5 to 5 h) and total RNA was isolated by TRIzol extraction. mRNA levels of Snail and β-actin were determined by real-time qrtPCR. Half-lives ($t½$) for the Snail gene were calculated by the following formula (Supplemental reference 18),

$$t_{1/2} = \left| \frac{\log(2)}{\log\{\mathrm{LOGEST}[(T_i, t_i):(T_f, t_f)]\}} \right|,$$

where the Microsoft Excel LOGEST function is used to estimate the slope of the points in the time course ranging from $t_i = 0$ to $t_f = 5$ h.

Measurement of circularity

The circularity assay was performed as described previously (Lee et al., 2015). Briefly, microscopic images were analyzed with Axiovision LE software (Release 4.8 version). Circularity was calculated using the formula $4\pi(\text{area}/\text{perimeter}^2)$. Values closer to 1.0 indicate a more circular cell morphology.

Statistical analysis

real-time qrtPCR and assays for mitochondrial respiration, Glc consumption, Lac production, and ATP production were performed at least in triplicate, and most experiments were repeated more than twice. Data were analyzed by the Student's *t*-test (unpaired, two-tailed), and results were expressed as mean ± SE. $p < 0.05$ was considered statistically significant.

The GEO accession numbers

The GEO accession number for the expression microarray data reported in this paper is GSE61009. Detailed information about the microarray data described in this study can be accessed from the NCBI GEO Web site (http://www.ncbi.nlm.nih.gov/geo) using this accession number.

SUPPLEMENTARY REFERENCES

1. Brabletz T. MiR-34 and SNAIL: another double-negative feedback loop controlling cellular plasticity/EMT governed by p53. Cell Cycle. 2012; 11: 215–216.
2. Chang CJ, Hsu CC, Chang CH, Tsai LL, Chang YC, Lu SW, Yu CH, Huang HS, Wang JJ, Tsai CH, Chou MY,

Yu CC, Hu FW. Let-7d functions as novel regulator of epithelial-mesenchymal transition and chemoresistant property in oral cancer. Oncol Rep. 2011; 26: 1003–1010.

3. Gill JG, Langer EM, Lindsley RC, Cai M, Murphy TL, Kyba M, Murphy KM. Snail and the microRNA-200 family act in opposition to regulate epithelial-to-mesenchymal transition and germ layer fate restriction in differentiating ESCs. Stem Cells. 2011; 29: 764–776.
4. Hu J, Guo H, Li H, Liu Y, Liu J, Chen L, Zhang J, Zhang N. MiR-145 regulates epithelial to mesenchymal transition of breast cancer cells by targeting Oct4. PLoS One. 2012; 7: e45965.
5. Kumarswamy R, Mudduluru G, Ceppi P, Muppala S, Kozlowski M, Niklinski J, Papotti M, Allgayer H. MicroRNA-30a inhibits epithelial-to-mesenchymal transition by targeting Snai1 and is downregulated in non-small cell lung cancer. Int J Cancer. 2012; 130: 2044–2053.
6. Liu Q, Liu T, Zheng S, Gao X, Lu M, Sheyhidin I, Lu X. HMGA2 is down-regulated by microRNA let-7 and associated with epithelial-mesenchymal transition in oesophageal squamous cell carcinomas of Kazakhs. Histopathology. 2014; 65:408–417.
7. Liu S, Kumar SM, Lu H, Liu A, Yang R, Pushparajan A, Guo W, Xu X. MicroRNA-9 up-regulates E-cadherin through inhibition of NF-kappaB1-Snail1 pathway in melanoma. J Pathol. 2012; 226: 61–72.
8. Liu Z, Liu H, Desai S, Schmitt DC, Zhou M, Khong HT, Klos KS, McClellan S, Fodstad O, Tan M. miR-125b functions as a key mediator for snail-induced stem cell propagation and chemoresistance. J Biol Chem. 2013; 288: 4334–4345.
9. Majid S, Dar AA, Saini S, Arora S, Shahryari V, Zaman MS, Chang I, Yamamura S, Tanaka Y, Deng G, Dahiya R. miR-23b represses proto-oncogene Src kinase and functions as methylation-silenced tumor suppressor with diagnostic and prognostic significance in prostate cancer. Cancer Res. 2012; 72: 6435–6446.
10. Moes M, Le Bechec A, Crespo I, Laurini C, Halavatyi A, Vetter G, Del Sol A, Friederich E. A novel network integrating a miRNA-203/SNAI1 feedback loop which regulates epithelial to mesenchymal transition. PLoS One. 2012; 7: e35440.
11. Qian P, Banerjee A, Wu ZS, Zhang X, Wang H, Pandey V, Zhang WJ, Lv XF, Tan S, Lobie PE, Zhu T. Loss of SNAIL regulated miR-128-2 on chromosome 3p22.3 targets multiple stem cell factors to promote transformation of mammary epithelial cells. Cancer Res. 2012; 72: 6036–6050.
12. Ru P, Steele R, Newhall P, Phillips NJ, Toth K, Ray RB. miRNA-29b suppresses prostate cancer metastasis by regulating epithelial-mesenchymal transition signaling. Mol Cancer Ther. 2012; 11: 1166–1173.
13. Wang FE, Zhang C, Maminishkis A, Dong L, Zhi C, Li R, Zhao J, Majerciak V, Gaur AB, Chen S, Miller SS. MicroRNA-204/211 alters epithelial physiology. Faseb J. 2010; 24: 1552–1571.
14. Xu Q, Sun Q, Zhang J, Yu J, Chen W, Zhang Z. Downregulation of miR-153 contributes to epithelial-mesenchymal transition and tumor metastasis in human epithelial cancer. Carcinogenesis. 2013; 34: 539–549.
15. Zhang JP, Zeng C, Xu L, Gong J, Fang JH, Zhuang SM. MicroRNA-148a suppresses the epithelial-mesenchymal transition and metastasis of hepatoma cells by targeting Met/Snail signaling. Oncogene. 2013; 33: 4069–4076.
16. Zhao X, Dou W, He L, Liang S, Tie J, Liu C, Li T, Lu Y, Mo P, Shi Y, Wu K, Nie Y, Fan D. MicroRNA-7 functions as an anti-metastatic microRNA in gastric cancer by targeting insulin-like growth factor-1 receptor. Oncogene. 2013; 32: 1363–1372.
17. Chang CJ, Chao CH, Xia W, Yang JY, Xiong Y, Li CW, Yu WH, Rehman SK, Hsu JL, Lee HH, Liu M, Chen CT, Yu D et al. p53 regulates epithelial-mesenchymal transition and stem cell properties through modulating miRNAs. Nat Cell Biol. 2011; 13: 317–323.
18. Geisberg JV, Moqtaderi Z, Fan X, Ozsolak F, Struhl K. Global analysis of mRNA isoform half-lives reveals stabilizing and destabilizing elements in yeast. Cell. 2014; 156: 812–824.

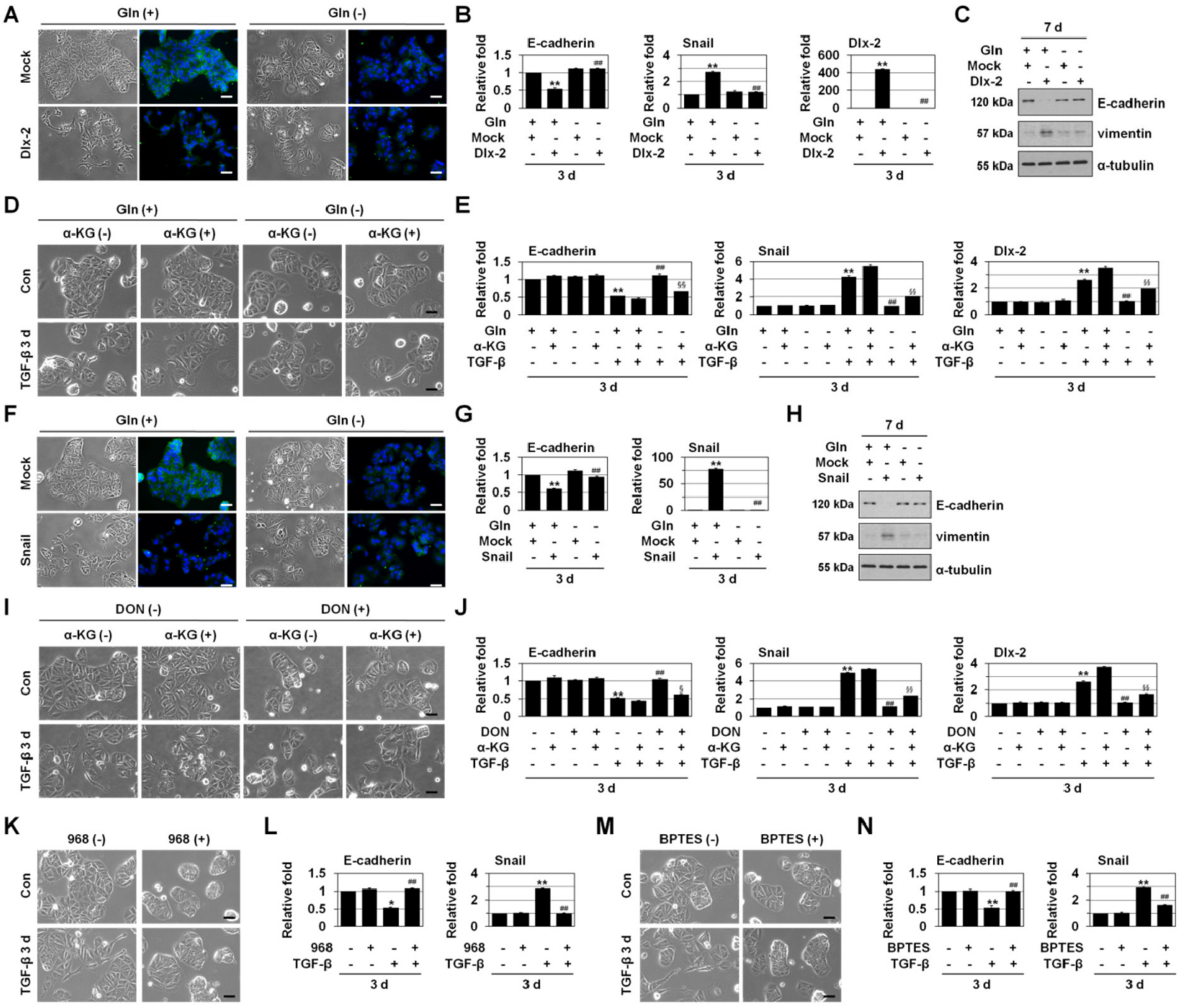

Supplementary Figure S1: Inhibition of Gln metabolism prevents EMT. A-C. MCF-7 cells were transfected with Dlx-2 and then cultured in complete or Gln-free medium. Cell morphology and E-cadherin expression (fluorescence in green) were analyzed by phase-contrast and fluorescence microscopy (A). The cells were also analyzed by real-time qrtPCR (B) and immunoblotting (C) using the indicated primers and antibodies. $^{**}p < 0.01$ versus Mock, $^{\#\#}p < 0.01$ versus complete medium. **D, E.** MCF-7 cells were treated with TGF-β and cultured in complete or Gln-free medium in the presence or absence of α-KG. Cell morphology was analyzed by phase-contrast microscopy (D). The cells were also analyzed by real-time qrtPCR using the indicated primers (E). $^{**}p < 0.01$ versus control, $^{\#\#}p < 0.01$ versus complete medium with TGF-β, $^{\S\S}p < 0.01$ versus TGF-β in Gln-free medium without α-KG. **F-H.** MCF-7 cells were transfected with Snail and then cultured in complete or Gln-free medium. Cell morphology and E-cadherin expression (fluorescence in green) were analyzed by phase-contrast and fluorescence microscopy (F). The cells were also analyzed by real-time qrtPCR (G) and immunoblotting (H) using the indicated primers and antibodies. $^{**}p < 0.01$ versus Mock, $^{\#\#}p < 0.01$ versus complete medium. **I, J.** MCF-7 cells were treated with DON and cultured in TGF-β-treated medium in the presence or absence of α-KG. Cell morphology was analyzed by phase-contrast microscopy (I). The cells were also analyzed by real-time qrtPCR using the indicated primers (J). $^{**}p < 0.01$ versus control, $^{\#\#}p < 0.01$ versus TGF-β without DON, $^{\S}p < 0.05$; $^{\S\S}p < 0.01$ versus TGF-β with DON and without α-KG. **K-N.** MCF-7 cells were treated with 968 (K, L) or BPTES (M, N) and cultured in TGF-β-treated medium. Cell morphology was analyzed by phase-contrast microscopy (K, M). The cells were also analyzed by real-time qrtPCR using the indicated primers (L, N). $^{*}p < 0.05$; $^{**}p < 0.01$ versus control, $^{\#\#}p < 0.01$ versus TGF-β without 968 or BPTES. All error bars represent SE. All scale bars represent 100 µm. For all immunoblotting images, cropped blots are shown.

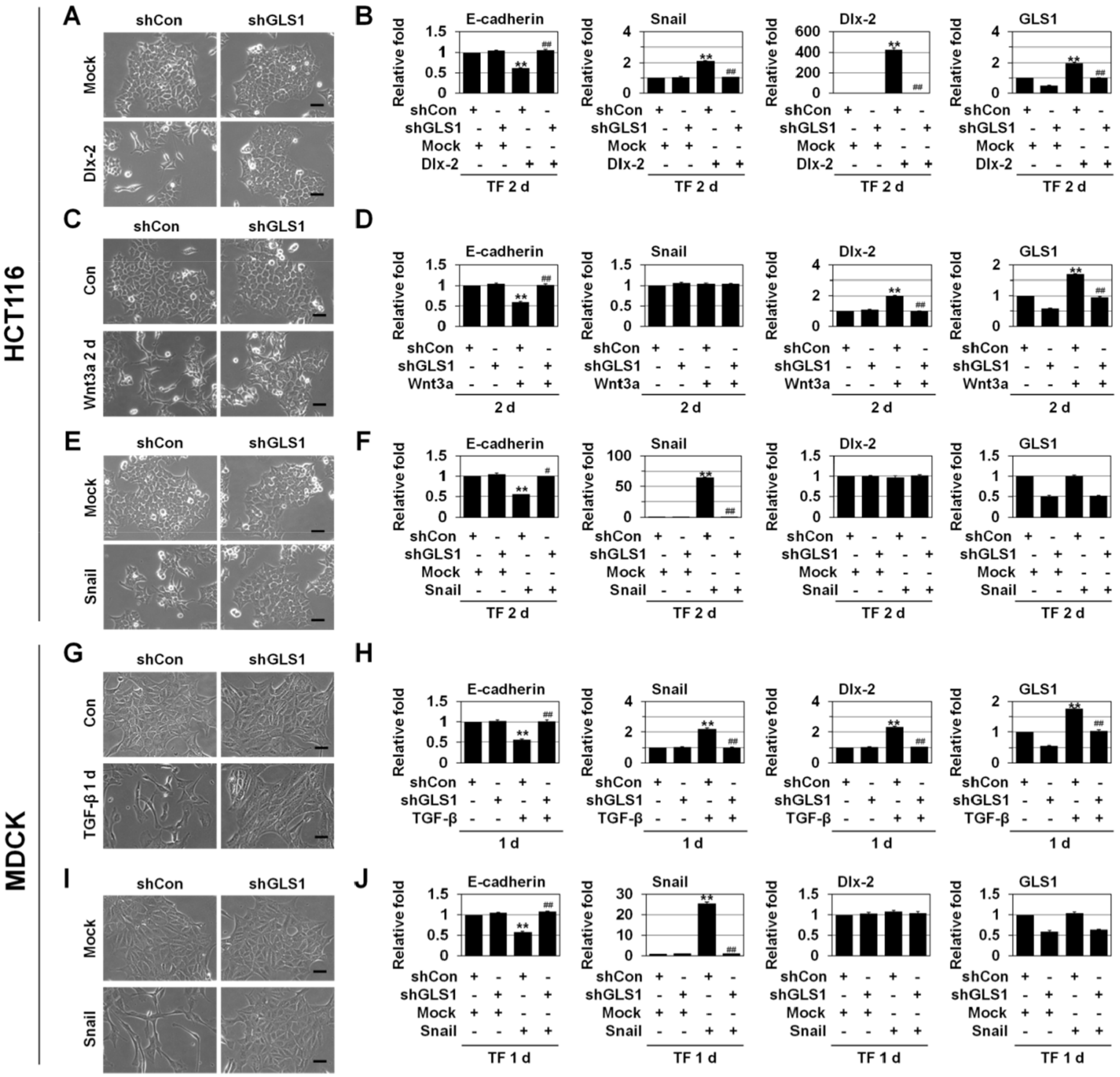

Supplementary Figure S2: Inhibition of Gln metabolism prevents EMT. A, B. HCT116 cells were co-transfected with Dlx-2 and shGLS1 and analyzed by phase-contrast microscopy for cell morphology (A). The cells were also analyzed by real-time qrtPCR using the indicated primers (B). $^{**}p < 0.01$ versus Mock, $^{\#\#}p < 0.01$ versus shCon. **C, D.** HCT116 cells transfected with shGLS1 and then treated with Wnt3a CM were analyzed by phase-contrast microscopy for cell morphology (C). The cells were also analyzed by real-time qrtPCR using the indicated primers (D). $^{**}p < 0.01$ versus untreated, $^{\#\#}p < 0.01$ versus shCon. **E, F.** HCT116 cells were co-transfected with Snail and shGLS1 and analyzed by phase-contrast microscopy for cell morphology (E). The cells were also analyzed by real-time qrtPCR using the indicated primers (F). $^{**}p < 0.01$ versus Mock, $^{\#}p < 0.05$; $^{\#\#}p < 0.01$ versus shCon. **G, H.** MDCK cells transfected with shGLS1 and then treated with TGF-β were analyzed by phase-contrast microscopy for cell morphology (G). The cells were also analyzed by real-time qrtPCR using the indicated primers (H). $^{**}p < 0.01$ versus untreated, $^{\#\#}p < 0.01$ versus shCon. **I, J.** MDCK cells were co-transfected with Snail and shGLS1 and analyzed by phase-contrast microscopy for cell morphology (I). The cells were also analyzed by real-time qrtPCR using the indicated primers (J). $^{**}p < 0.01$ versus Mock, $^{\#\#}p < 0.01$ versus shCon. All error bars represent SE. All scale bars represent 100 μm.

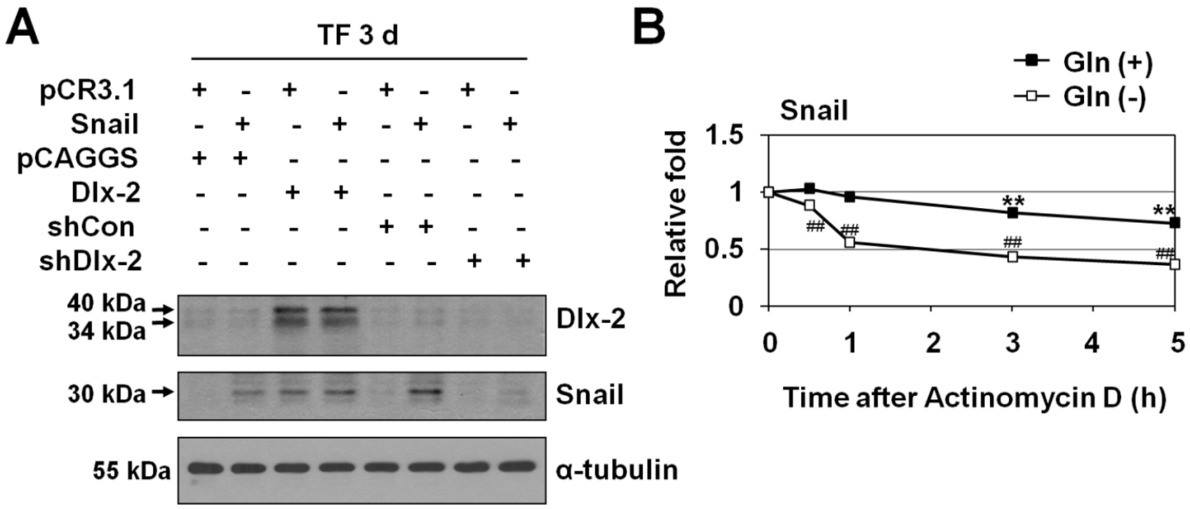

Supplementary Figure S3: shDlx-2 or Gln metabolism inhibition decrease Snail mRNA levels. **A.** MCF-7 cells were co-transfected with Snail and Dlx-2 or with Snail and shDlx-2 and analyzed by immunoblotting for Dlx-2, Snail and α-tubulin. **B.** MCF-7 cells were transfected with Snail for 18 h, rinsed 3 times, and then cultured in complete or Gln-free medium for the chase period (for 0.5-5 h) with addition of actinomycin D. Values were normalized to β-actin. $^{**}p < 0.01$ versus 0 h, $^{\#\#}p < 0.01$ versus complete medium. All error bars represent SE. For all immunoblotting images, cropped blots are shown.

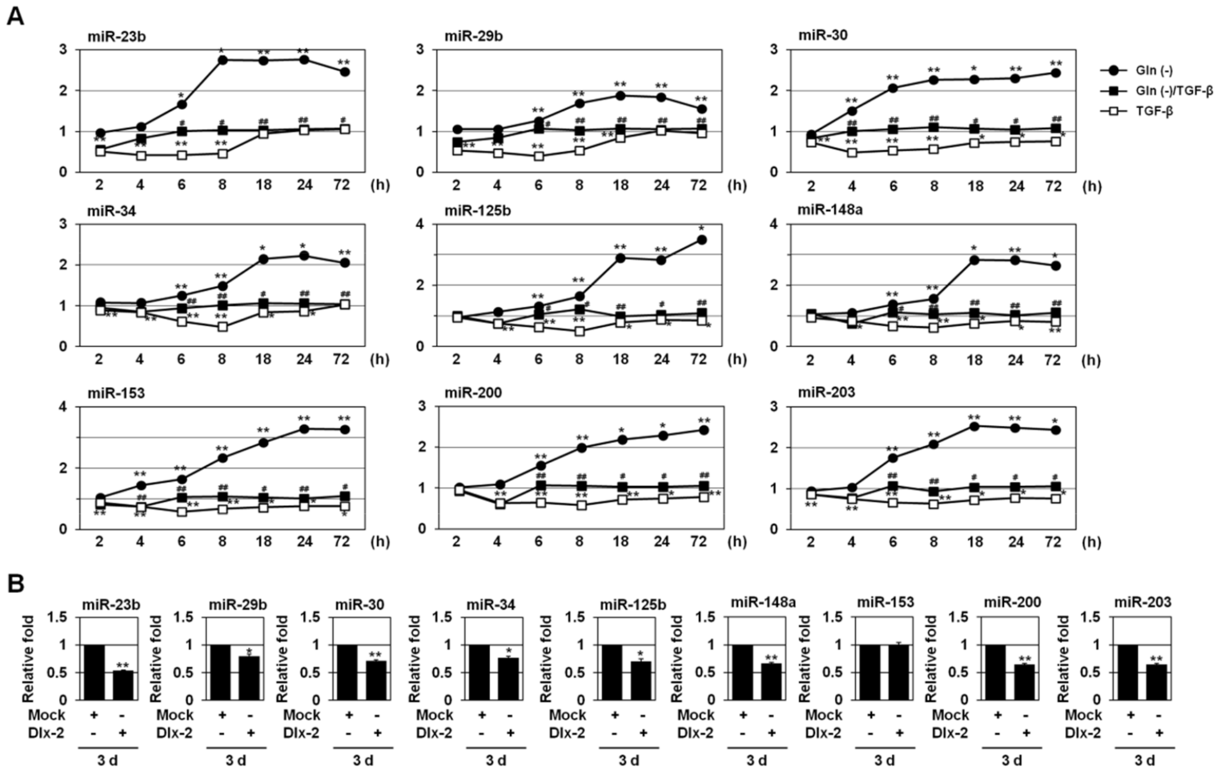

Supplementary Figure S4: TGF-β or Dlx-2 reduced the expression of the Snail-targeting miRNA. A. MCF-7 cells were cultured in complete or Gln-free medium and then treated with TGF-β for the indicated times. miRNA levels were analyzed by real-time qrtPCR. Expression of each miRNA was normalized to RNU6. *$p < 0.05$; **$p < 0.01$ versus control, $^{\#}p < 0.05$; $^{\#\#}p < 0.01$ versus Gln-free medium without TGF-β. **B.** MCF-7 cells transfected with Dlx-2 were analyzed by real-time qrtPCR for Snail-targeting miRNA expression. *$p < 0.05$; **$p < 0.01$ versus Mock. All error bars represent SE.

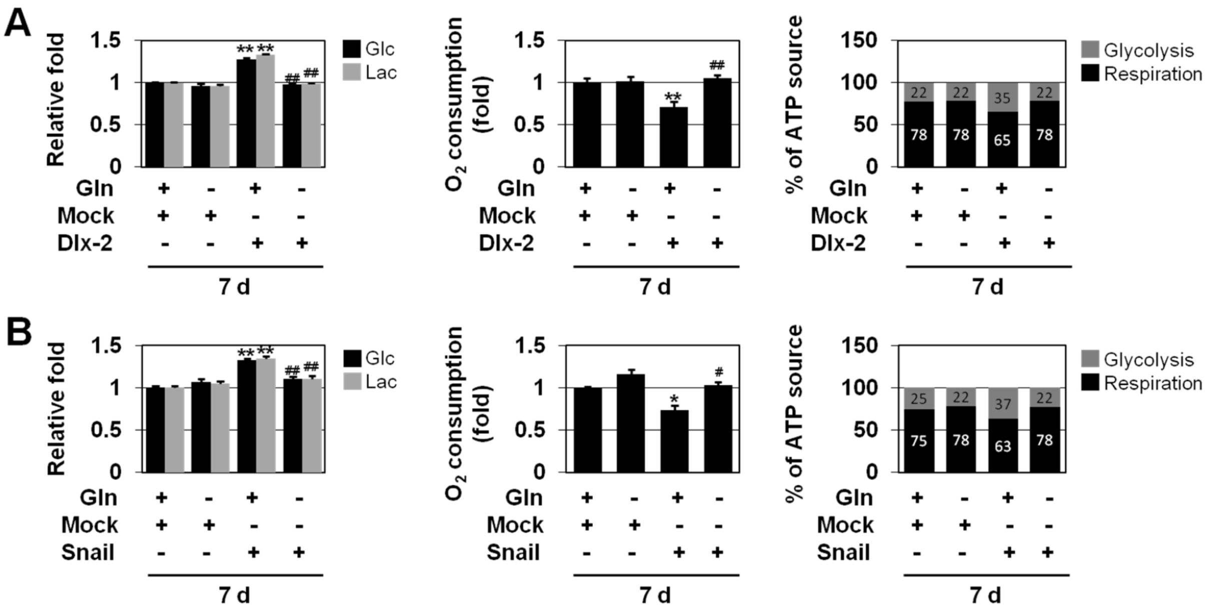

Supplementary Figure S5: Gln metabolism is linked to Dlx-2/Snail-induced glycolytic switch and mitochondrial repression. A, B. Glc consumption, Lac production, mitochondrial respiration, and ATP source were analyzed in MCF-7 cells transfected with Dlx-2 (A) or Snail (B), and then cultured in complete or Gln-free medium. *$p < 0.05$; **$p < 0.01$ versus Mock, $^{\#}p < 0.05$; $^{\#\#}p < 0.01$ versus complete medium. The amount of ATP produced by aerobic respiration (black bars) and glycolysis (gray bars) was calculated by measuring oxygen consumption and Lac production in the cells (right panels in A and B). All error bars represent SE.

Supplementary Table S1: shRNA target sequences used in this paper

Gene	target sequence 5' to 3'
Con shRNA	AATTCTCCGAACGTGTCACGT
Snail shRNA	GCGAGCTGCAGGACTCTAA
Dlx-2 shRNA	TTCGGATAGTGAACGGGAA
GLS1 shRNA #1	CCCAGGTTGAAAGAGTGTATGGATA
GLS1 shRNA #2	GCACAGACATGGTTGGTATAT
p53 shRNA	GACTCCAGTGGTAATCTACTG
Smad2 shRNA	AACAAACCAGGTCTCTTGATG
Smad3 shRNA	CAGCACATAATAACTTGGACCTGCA
Smad4 shRNA	CGAGTTGTATCACCTGGAA
β-catenin shRNA	GATAAAGGCTACTGTTGGA
Axin1 shRNA	GCCGACCTTAAATGAAGATGA
Axin2 shRNA	AGACGATACTGGACGATCA
TCF4 shRNA	GCCTTTCACTTCCTCCGATTA

Supplementary Table S2: Primers used in this paper for real-time qrtPCR and ChIP assays

Gene			target sequence 5' to 3'	Annealing °C
**qrtPCR**				
β-actin	NM_001101.3	sense	ACTCTTCCAGCCTTCCTTCC	
		antisense	TGTTGGCGTACAGGTCTTTG	
Snail	NM_005985	sense	ATCGGAAGCCTAACTACAGC	55
		antisense	CAGAGTCCCAGATGAGCATT	
Dlx-2	NM_004405	sense	GCA CATGGGTTCCTACCAGT	62
		antisense	ACTTTCTTTGGCTTCCCGTT	
E-cadherin	NM_004360	sense	GATTTTGAGGCCAAGCAGCA	55
		antisense	AGATGGGGGCTTCATTCACA	
GLS1	NM_001256310.1	sense	CCGGTCGCGGCAATCCTAGC	62
		antisense	GTCTGTGGTGGGGCGGTGAG	
p53	NM_000546.5	sense	CCTCACCATCATCACACTGG	55
		antisense	GCTCTCGGAACATCTCGAAG	
**ChIP assay**				
GLS1 #1 (for D1/D2)		sense	TGTGGATCTACTCCATTTAAACCT	60
		antisense	GGACCTGTGGAAAAACAGCC	
GLS1 #2 (for D3)		sense	TGCTTTCAAGCAAATAGCGTT	60
		antisense	TGCGGAATCCAGAATACGAC	
GLS1 #3 (for D4)		sense	CCCGCTTCACACGTCAGTTTGA	60
		antisense	GCTGCGATTGGCTCAAATCCTC	

Supplementary Table S3: Putative Dlx-2 binding sites in promoter region

Gene	Dlx-2 binding sites	Positions from transcription start site
GLS1	D1	$^{-1036}$ TAAT $^{-1033}$
	D2	$^{-989}$ TAAT $^{-986}$
	D3	$^{-733}$ TAAT $^{-730}$
	D4	$^{-123}$ TAAT $^{-120}$
